# Cost-Sharing and Buprenorphine Prescription Dispensing

**DOI:** 10.1001/jamahealthforum.2025.1913

**Published:** 2025-07-03

**Authors:** Thuy D. Nguyen, Rena M. Conti, Pooja Lagisetty, Amy S. B. Bohnert, Ushapoorna Nuliyalu, Kao-Ping Chua

**Affiliations:** 1 Department of Health Management and Policy, University of Michigan School of Public Health, Ann Arbor; 2 Department of Markets, Public Policy, and Law, Questrom School of Business, Boston University, Boston, Massachusetts; 3 Department of Internal Medicine, University of Michigan Medical School, Ann Arbor; 4 Department of Anesthesiology, University of Michigan Medical School, Ann Arbor; 5 Susan B. Meister Child Health Evaluation and Research Center, Department of Pediatrics, University of Michigan Medical School, Ann Arbor

## Abstract

This cross-sectional study estimates the association between cost-sharing and buprenorphine dispensing among commercially insured and Medicare-insured patients.

## Introduction

Increasing dispensing of buprenorphine is crucial to slow the US opioid epidemic. Whether decreasing cost-sharing for buprenorphine can achieve this goal is unclear, as studies on the association between cost-sharing and dispensing have had mixed results and have been prone to unobserved confounding.^1,2,3,4^ We estimated the association between cost-sharing and buprenorphine dispensing among commercially insured and Medicare-insured patients by exploiting the abrupt increase in cost-sharing at the beginning of the calendar year, when deductibles typically reset in private and Medicare plans.

## Methods

This cross-sectional study used deidentified data; therefore, the University of Michigan Institutional Review Board exempted it from review and waived informed consent. We followed the STROBE reporting guideline for cross-sectional studies.

We analyzed the 2021-2022 IQVIA Formulary Impact Analyzer (IQVIA Inc), which reports pharmacy transactions for 63% of prescriptions from US pharmacies. This database includes transaction records (hereinafter, claims) for all prescriptions received by contributing pharmacies regardless of whether prescriptions were dispensed.

We identified claims for 1 of 5 immediate-release buprenorphine products approved to treat opioid use disorder among commercially insured and Medicare-insured patients. We limited our analysis to claims occurring 60 days before through 60 days after January 1, 2022 (November 2, 2021, through March 1, 2022). Data were analyzed from November 11, 2023, to November 22, 2024.

The exposure was cost-sharing per 30-day supply of buprenorphine. The outcome was prescription abandonment, defined as a lack of prescription dispensing within 14 days of the date the pharmacy received the prescription. We used local linear regression models to assess for abrupt discontinuities in cost-sharing and abandonment on January 1, 2022. To estimate the association between cost-sharing and abandonment, we conducted a 2-stage least-squares instrumental variable analysis in which January 1, 2022, was the instrument for cost-sharing. We used 2-sided hypothesis tests with α = .05 and conducted analyses using the rdrobust package in Stata, version 18.1/MP (StataCorp LLC). The eMethods in Supplement 1 provides additional details.

## Results

The study included 674 249 claims for commercially insured patients (mean [SD] age, 42.5 [11.1] years) and 362 748 claims for Medicare-insured patients (mean [SD] age, 56.0 [12.5] years) . These prescriptions were for 215 041 unique commercially insured patients (36.9% female, 63.1% male) and 100 092 unique Medicare-insured patients (49.0% female, 51.0% male).

**Figure 1.**
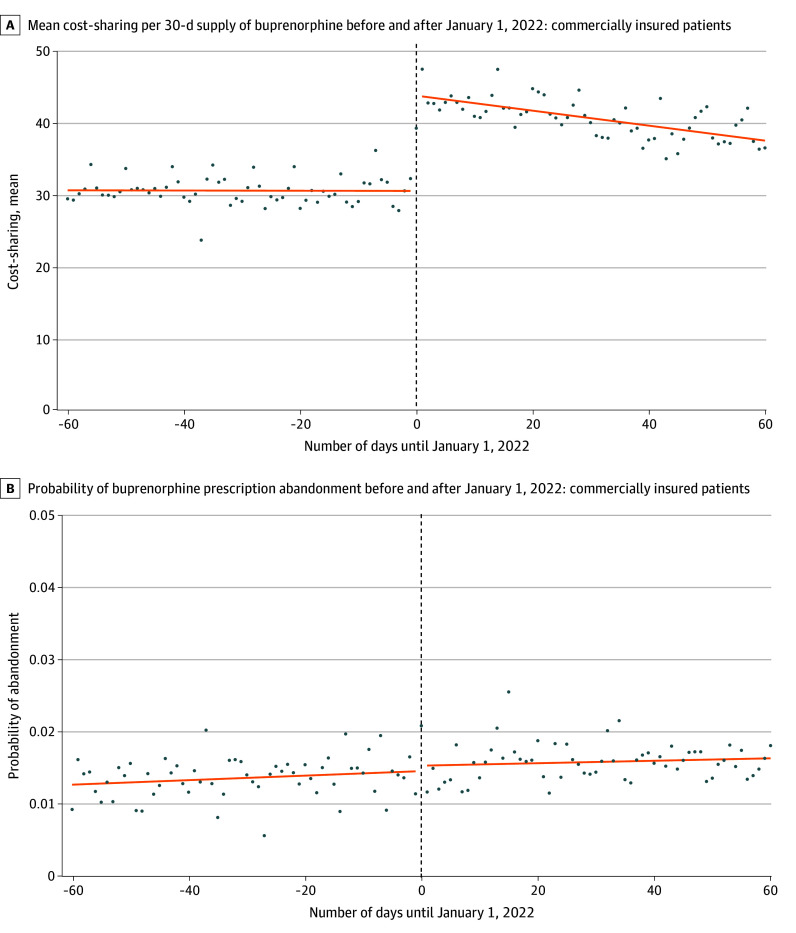
Cost-Sharing and Probability of Prescription Abandonment Among Buprenorphine Claims for Commercially Insured Patients A, Each dot represents the mean cost-sharing across buprenorphine prescription claims on the date in question. B, Each dot represents the proportion of buprenorphine prescription claims on the date in question that were abandoned. Abandonment is defined as a lack of prescription dispensing within 14 days of receipt by the pharmacy.

**Figure 2.**
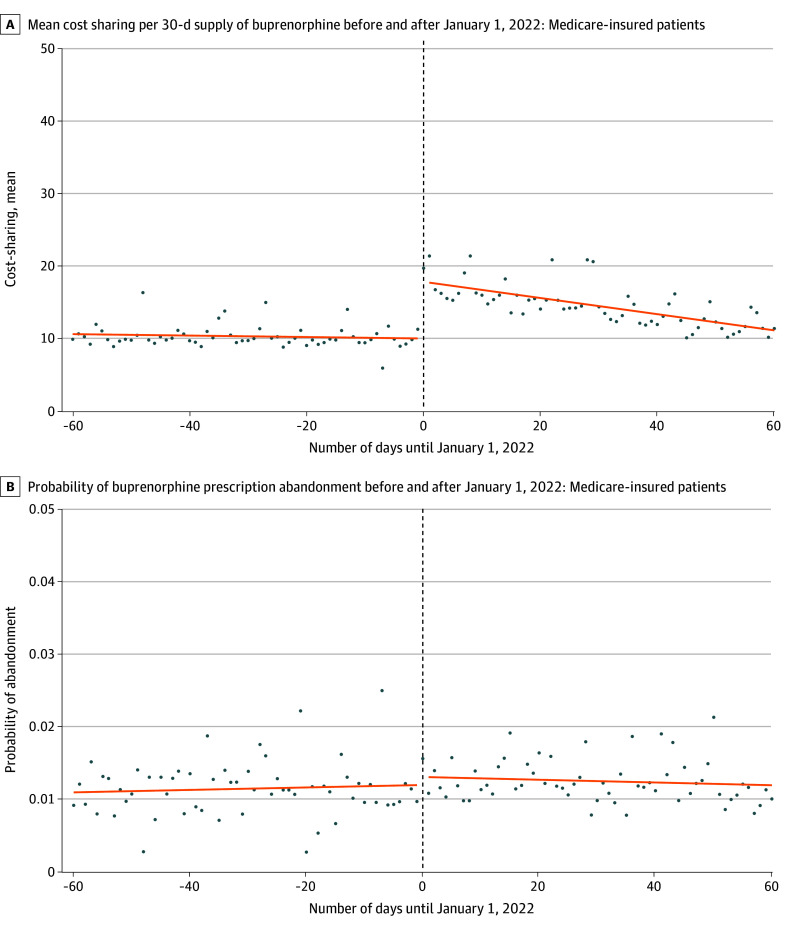
Cost-Sharing and Probability of Prescription Abandonment Among Buprenorphine Claims for Medicare-Insured Patients A, Each dot represents the mean cost-sharing across buprenorphine prescription claims on the date in question. B, Each dot represents the proportion of buprenorphine prescription claims on the date in question that were abandoned. Abandonment is defined as a lack of prescription dispensing within 14 days of receipt by the pharmacy. Medicare-insured patients include those covered by traditional Medicare or Medicare Advantage plans.

Among claims for commercially insured patients, the mean cost-sharing per 30-day supply increased $13.38 (95% CI, $12.21-$14.54) on January 1, 2022, but the probability of abandonment did not change (−0.1 percentage points [pp]; 95% CI, −0.3 to 0.1). We estimated that a $10 increase in cost-sharing per 30-day supply would not be associated with a change in abandonment (−0.1 pp; 95% CI, −0.2 to 0.1) (Figure 1). Among claims for Medicare-insured patients, the mean cost-sharing per 30-day supply increased by $6.98 (95% CI, $6.03-7.93), but the probability of abandonment did not change (0.2 pp; 95% CI, −0.02 to 0.5 pp). We estimated that a $10 increase in cost-sharing per 30-day supply would be associated with an increase of 0.3 pp (95% CI, 0.02-0.6 pp) for probability of abandonment (Figure 2).

## Discussion

Our findings suggest that an increase of $10 in cost-sharing per 30-day supply of buprenorphine would be associated with no immediate increase in prescription abandonment among commercially insured patients and a small increase among Medicare-insured patients. Therefore, cost-sharing may not be an important factor in prescription abandonment among privately insured and Medicare-insured patients. Thus, any efforts to eliminate cost-sharing should be coupled with interventions addressing other barriers to dispensing (eg, stigma and lack of access to prescribers).^5,6^

Study limitations include the inability to differentiate between prescriptions for pain vs opioid use disorder. Additionally, our findings may not generalize beyond the study cutoff date or to patients who do not have deductibles. Finally, the findings may not generalize to patients’ decisions on whether to seek the health care required to receive buprenorphine prescriptions (eg, outpatient visits). Future studies should investigate whether high-cost sharing is associated with decreased receipt of this care.

## Notes

Supplement 1. **eMethods. **Methodological Details
eReferences

Supplement 2.
Data Sharing Statement

## References

[1] Dunphy C, Peterson C, Zhang K, Jones CM. Do out-of-pocket costs influence retention and adherence to medications for opioid use disorder? Drug Alcohol Depend. 2021;225(108784):108784. doi:10.1016/j.drugalcdep.2021.108784 34049104 PMC8314254

[2] Leech AA, McNeer E, Roberts AW, . Buprenorphine out-of-pocket costs and discontinuation in privately insured adults with opioid use disorder. JAMA Intern Med. 2023;183(9):1023-1026. doi:10.1001/jamainternmed.2023.2826 37548972 PMC10407758

[3] McClellan C, Fingar KR, Ali MM, Olesiuk WJ, Mutter R, Gibson TB. Price elasticity of demand for buprenorphine/naloxone prescriptions. J Subst Abuse Treat. 2019;106:4-11. doi:10.1016/j.jsat.2019.08.001 31540610

[4] Chua KP, Conti RM, Lagisetty P, Bohnert ASB, Nuliyalu U, Nguyen TD. Association between cost-sharing and buprenorphine prescription abandonment. J Gen Intern Med. 2024;39(12):2160-2168. doi:10.1007/s11606-024-08819-2 38888865 PMC11347500

[5] Mackey K, Veazie S, Anderson J, Bourne D, Peterson K. Barriers and facilitators to the use of medications for opioid use disorder: a rapid review. J Gen Intern Med. 2020;35(suppl 3):954-963. doi:10.1007/s11606-020-06257-4 33145687 PMC7728943

[6] Stein BD, Saloner B, Schuler MS, Gurvey J, Sorbero M, Gordon AJ. Concentration of patient care among buprenorphine-prescribing clinicians in the US. JAMA. 2021;325(21):2206-2208. doi:10.1001/jama.2021.4469 34061152 PMC8170540

