# Supplementary material for: Cost-Sharing and Buprenorphine Prescription Dispensing
Source: JAMA Health Forum. 2025 Jul 3;6(7):e251913. doi: 10.1001/jamahealthforum.2025.1913 (PMC12232175; doi:10.1001/jamahealthforum.2025.1913)
Supplement: Supplement 1. — eMethods. Methodological Details eReferences [file jamahealthforum-e251913-s001.pdf]

Supplemental Online Content

Nguyen TD, Conti RM, Lagisetty P, Bohnert ASB Nuliyalu U, Chua KP. Cost-sharing and buprenorphine prescription dispensing. *JAMA Health Forum*. Published online July 3, 2025. doi:10.1001/jamahealthforum.2025.1913

eMethods. Methodological Details

eReferences

This supplemental material has been provided by the authors to give readers additional information about their work.

© 2025 Nguyen TD et al. *JAMA Health Forum*.

eMethods. Methodological Details

Sample. Analyses were conducted at the level of claims. We identified claims for one of 5 immediate-release buprenorphine products approved to treat opioid use disorder among U.S. commercially insured and Medicare patients in 2021-2022. The 5 products included generic buprenorphine tablets, generic and branded buprenorphine/naloxone film, and generic and branded buprenorphine/naloxone tablets. We did not include extended-release injectable buprenorphine products administrated by a health care professional during visits,[1] as these products often are covered under a patient's medical rather than pharmacy benefit and therefore are incompletely captured in the IQVIA Formulary Impact Analyzer. We also did not include claims for buprenorphine prescriptions written by veterinarians or those that were rejected by insurers, as the IQVIA Formulary Impact Analyzer does not report cost-sharing for these claims. Among claims meeting inclusion criteria, we excluded those for which the transaction date did not occur during the 60 days before through 60 days after January 1, 2022 (i.e., November 2, 2021, through March 1, 2022). We limited to this 60-day "bandwidth" to allow use of local linear regression models.[2] Additionally, we excluded claims with missing data for cost-sharing and duplicate claims.

Sample inclusion and exclusion criteria. Among 5,462,246 buprenorphine claims during 2021-2022 that had commercial method of payment and met inclusion criteria, 12,989 were excluded owing to missing data on cost-sharing, 686,114 were excluded during the de-duplication procedure, and 685,783 were excluded because they were not standalone claims. Of the remaining 4,090,379 claims, 674,249 occurred within 60 days of January 1, 2022.

Among 2,823,018 claims during 2021-2022 that had Medicare method of payment and met inclusion criteria, 7,007 were excluded owing to missing data on cost-sharing, 331,931 were excluded during the de-duplication procedure, and 325,363 were excluded because they were not standalone claims. Of the remaining 2,158,717 claims, 362,748 occurred within 60 days of January 1, 2022.

Standalone claims and deduplication procedure. The IQVIA Formulary Impact Analyzer follows a prescription over its lifecycle, meaning that a given prescription could result in multiple claims (transaction records). The database does not include a unique prescription identifier. In our analysis, we applied a procedure to allow facilitate interpretation of claims as prescriptions and to increase the uniformity of the sample. First, we deduplicated claims by the following combination of variables: patient identifier, provider identifier, national drug code, date of writing, days supplied, quantity, and dispense as written code. Second, we limited to "standalone claims." Per IQVIA's standard business rules, standalone claims are those with no other reversed or rejected claims (i.e., unsuccessful fill attempts of the medication in question) during a 14-day "look-forward period" and a 60-day "look-back period."

Study design. We conducted a regression discontinuity analysis. In this strong quasi-experimental design, the effect of an exposure on an outcome is estimated by comparing the magnitude of the discontinuity in the exposure and outcome at some cutoff of a "running variable" that predicts either the receipt or magnitude of the treatment. If no other factors affecting the outcome change abruptly at the cutoff, regression discontinuity designs can provide

© 2025 Nguyen TD et al. *JAMA Health Forum*.

plausibly unbiased effect estimates. Although the exposure in regression discontinuity analyses is often binary, continuous exposures – such as the one used in this study – can be accommodated using a so-called “fuzzy” regression discontinuity approach.[2]

The exposure was cost-sharing, standardized to a 30-day supply to enhance result interpretation, similar to prior studies.[3,4] For example, if cost-sharing for a prescription for a 15-day supply of buprenorphine were $10, cost-sharing per 30-day supply would be $20. We applied this transformation to account for the differing durations of buprenorphine prescriptions. The outcome was prescription abandonment. Following IQVIA’s standard business rules, abandonment was defined as the lack of prescription dispensing within 14 days of the date the pharmacy received the prescription. The running variable was the number of days before or after the cutoff (January 1, 2022).

Statistical analyses. Our analysis followed the general approach of a previous regression discontinuity analysis we conducted to estimate the association between cost-sharing and naloxone dispensing.[5] First, we fitted local regression models with one-day bins and a triangular kernel to assess for abrupt discontinuities in cost-sharing and prescription abandonment on January 1, 2022. Second, we conducted a two-stage least squares instrumental variable analysis in which January 1, 2022 was used as the instrument for cost-sharing. The point estimate from this analysis equaled the magnitude of the discontinuity in probability of abandonment divided by the magnitude of discontinuity in cost-sharing per 30-day supply. To facilitate interpretation, we multiplied this point estimate and the 95% confidence interval by $10 so that we could report the effect of a $10 increase in cost-sharing on the probability of abandonment. All analyses were conducted separately among claims for commercially insured and Medicare patients.

We employed robust biased-corrected inference in all regression discontinuity analyses.[6] We used two-sided hypothesis tests with $\alpha=0.05$ and conducted analyses using the rdrobust package in Stata 18.1/MP (StataCorp, College Station, TX).[7]

Two-stage least squares analysis in a fuzzy regression discontinuity framework. In our study, we used the rdrobust package to estimate local linear regression models with robust bias-corrected inference to calculate the discontinuities in buprenorphine cost-sharing per 30-day supply and abandonment on January 1, 2022. Although we could have calculated the point estimate of the effect of cost-sharing per 30-day supply on abandonment by calculating the ratio between the two discontinuities, we could not have generated confidence intervals. Therefore, we made reference on the effect of cost-sharing on abandonment by using a so-called “local two-stage least squares” estimation in a fuzzy regression discontinuity framework in which January 1, 2022 was the instrument for cost-sharing per 30-day supply. A parametric fuzzy regression discontinuity analysis is equivalent to the following two-step estimation. In the first stage, cost-sharing was modeled as a function of the number of days until January 1, 2022, as well as an indicator for this date. In the second stage, the probability of abandonment was modeled as a function of the predicted values from the first-stage regression.

This two-stage least squares analysis assumed that January 1, 2022 is strongly associated with cost-sharing per 30-day supply. Evidence for this assumption comes from the abrupt increase in cost-sharing per 30-day supply on January 1. Additionally, the F statistics for the first stage were

© 2025 Nguyen TD et al. *JAMA Health Forum*.

1,359 and 384 for commercially insured and Medicare patients, respectively, suggesting the instrument is strong.

eReferences

1. US Food & Drug Administration. FDA approves first once-monthly buprenorphine injection, a medication-assisted treatment option for opioid use disorder. November 30, 2017. Accessed November 10, 2024. https://www.fda.gov/news-events/press-announcements/fda-approves-first-once-monthly-buprenorphine-injection-medication-assisted-treatment-option-opioid

2. Lee DS, Lemieux T. Regression Discontinuity designs in economics. *J Econ Lit*. 2010;48(2):281-355.

3. Chernew M, Gibson TB, Yu-Isenberg K, Sokol MC, Rosen AB, Fendrick AM. Effects of increased patient cost sharing on socioeconomic disparities in health care. *J Gen Intern Med*. 2008;23(8):1131-1136.

4. Dusetzina SB, Winn AN, Abel GA, Huskamp HA, Keating NL. Cost sharing and adherence to tyrosine kinase inhibitors for patients with chronic myeloid leukemia. *J Clin Oncol*. 2014;32(4):306-311.

5. Chua KP, Conti RM, Lagisetty P, Bohnert AS, He S, Nguyen TD. Association between cost sharing and naloxone prescription dispensing. *JAMA*. 2024;332(2):124-132.

6. Calonico S, Cattaneo MD, Farrell MH. Optimal bandwidth choice for robust bias-corrected inference in regression discontinuity designs. *Econom J*. 2020;23(2):192-210.

7. Calonico S, Cattaneo MD, Farrell MH, Titiunik R. Rdrobust: Software for regression-discontinuity designs. *Stata J*. 2017;17(2):372-404.

8. McCrary J. Manipulation of the running variable in the regression discontinuity design: A density test. *J Econom*. 2008;142(2):698-714.

9. Cattaneo MD, Jansson M, Ma X. rddensity: Manipulation testing based on density discontinuity. *The Stata Journal (ii)*. Published online 2016. https://rpkg.net/packages/rddensity/reference/rddensity-package.ob

© 2025 Nguyen TD et al. *JAMA Health Forum*.
