# Supplementary material for: Cost-Sharing and Buprenorphine Prescription Dispensing
Source: JAMA Health Forum. 2025 Jul 3;6(7):e251913. doi: 10.1001/jamahealthforum.2025.1913 (PMC12232175; doi:10.1001/jamahealthforum.2025.1913)
Supplement: Supplement 2. — Data Sharing Statement [file jamahealthforum-e251913-s002.pdf]

Data Sharing Statement

Nguyen. Cost-Sharing and Buprenorphine Prescription Dispensing. *JAMA Health Forum*. Published July 03, 2025. doi:10.1001/jamahealthforum.2025.1913

Data
Data available: No
Explanation for why data not available: IQVIA data are proprietary and cannot be shared.
